# Increased male fertility using fertility-related biomarkers

**DOI:** 10.1038/srep15654

**Published:** 2015-10-22

**Authors:** Woo-Sung Kwon, Md Saidur Rahman, Do-Yeal Ryu, Yoo-Jin Park, Myung-Geol Pang

**Affiliations:** 1 Department of Animal Science & Technology, Chung-Ang University, Anseong, Gyeonggi-Do 456-756, Korea

## Abstract

Conventional semen analyses are used to evaluate male factor fertility/infertility in humans and other animals. However, their clinical value remains controversial. Therefore, new tools that more accurately assess male fertility based on sperm function and fertilization mechanism are of interest worldwide. While protein markers in spermatozoa that might help differentiate fertile and infertile sperm have been identified, studies are in their infancy, and the markers require validation in field trials. In the present study, to discover more sensitive biomarkers in spermatozoa for predicting male fertility, we assessed protein expression in capacitated spermatozoa. The results demonstrated that cytochrome b-c1 complex subunit 2 (UQCRC2) was abundantly expressed in high-litter size spermatozoa (>3-fold). On the other hand, equatorin, beta-tubulin, cytochrome b-c1 complex subunit 1 (UQCRC1), speriolin, Ras-related protein Rab-2A (RAB2A), spermadhesin AQN-3, and seminal plasma sperm motility inhibitor were abundantly expressed in low-litter size spermatozoa (>3-fold). Moreover, RAB2A and UQCRC1 expression negatively correlated with litter size, while UQCRC2 expression positively correlated with litter size. Finally, the putative biomarkers predicted litter size in field trials. Our study suggests that biomarkers present in spermatozoa after capacitation can help differentiate superior male fertility from below-average fertility with high sensitivity.

Worldwide, the prognosis and diagnosis of male fertility are important for the reproduction of economic animals. About half of human pregnancy failures can be attributed to decreased male fertility or male factor infertility^1^,^2^,^3^. The same is true in animal breeding systems. Although artificial insemination (AI) has been used to breed high-quality livestock, only 50% of such inseminations result in successful full-term pregnancies^4^,^5^. This pregnancy failure gives rise to huge economic losses. To evaluate sperm fertility, semen analyses, such as the sperm morphology test^6^, motility test^7^, swelling/eosin test^8^ and penetration assay^9^, have been developed for use in humans and other animals. Although these tools provide initial quantitative information on semen, their clinical value in predicting fertility is debated^10^. Therefore, new analysis tools based on sperm function and fertilization mechanism are needed.

After ejaculation, mammalian spermatozoa inhabitate the female genital tract for a considerable period of time, during which they undergo necessary modifications, including capacitation^11^. Capacitation is a cascade of biochemical events involving protein kinase A-dependent protein phosphorylation of sperm proteins, cholesterol efflux, changes in intracellular ion (Ca^2+^, Na^+^, K^+^, Cl^−^, and HCO_3_^−^) concentrations, and motility^11^,^12^,^13^. Kwon *et al.*^14^ have reported that the phosphorylation of sperm proteins plays a key role in regulating sperm capacitation. Thus, protein phosphorylation might be useful as a capacitation index. Following capacitation, pre-existing spermatozoa proteins are altered and new proteins are expressed to regulate the cellular processes that lead to the acquisition of mature functions. This process must occur before fertilization of the oocyte^12^,^13^. Several studies have identified differentially expressed proteins in mammalian spermatozoa associated with capacitation or litter size^15^,^16^,^17^,^18^,^19^,^20^. However, a single event is only partially able to represent the state of male fertility.

To develop highly sensitive biomarkers for the evaluation of male fertility, comprehensive studies of capacitated spermatozoa that yield high- and low-litter sizes in boar were performed. First, to induce spermatozoa capacitation, boar spermatozoa were exposed to capacitation medium. Sperm kinematics, morphology, and tyrosine phosphorylation levels were then assessed using computer-assisted sperm analysis (CASA), combined Hoechst 33258/chlortetracycline (H33258/CTC) fluorescence assessment of capacitation status, and western blotting, respectively. Second, a two-dimensional gel electrophoresis (2-DE) proteomics approach was used to identify differentially expressed proteins using electrospray ionization (ESI)-MS/MS and a MASCOT search. Differential protein expression was confirmed with western blotting, and enzyme-linked immunosorbent assay (ELISA) was used to determine the relationship between protein expression at capacitation and fertility.

## Results

### Spermatozoa motion kinematics, capacitation status, and tyrosine phosphorylation status following the induction of capacitation

**Figure 1:**
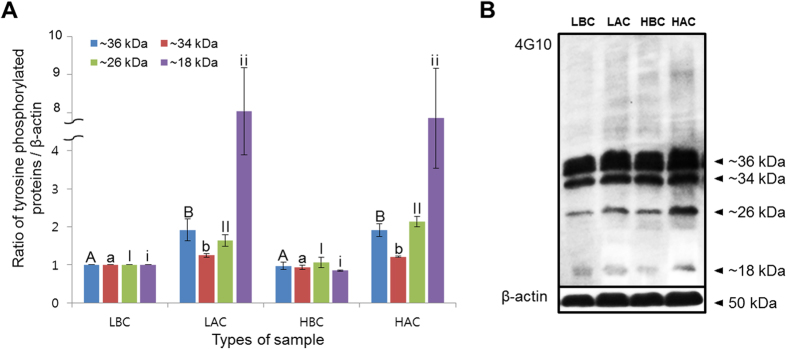
Levels of tyrosine phosphorylation in high- and low-litter size spermatozoa following capacitation. (**A**) Ratios of tyrosine phosphorylated protein (optical density [OD] × mm)/β-actin (OD × mm) (blue bar: ~36 kDa, red bar: ~34 kDa, green bar: ~26 kDa, purple bar: ~18 kDa; LBC: low-litter size spermatozoa before capacitation, LAC: low-litter size spermatozoa after capacitation, HBC: high-litter size spermatozoa before capacitation, HAC: high-litter size spermatozoa after capacitation). Data represent the mean ± SEM, n = 3. Values with different superscripts (^A,B, a, b, I, II, i, ii^) were significantly different (*P* < 0.05). (**B**) Tyrosine phosphorylated proteins were probed with anti-phosphotyrosine (4G10) antibody. Lane 1: low-litter size spermatozoa before capacitation, lane 2: low-litter size spermatozoa after capacitation, lane 3: high-litter size spermatozoa before capacitation, lane 4: high-litter size spermatozoa after capacitation.

Sperm motion kinematics and morphological changes related to capacitation were evaluated with CASA and H33258/CTC staining. Hyperactivation, curvilinear velocity, the mean amplitude of head lateral displacement, linearity, wobble, acrosome-reacted spermatozoa, and capacitated spermatozoa increased after the induction of capacitation regardless of the litter size. Non-capacitated spermatozoa decreased after capacitation regardless of litter size (*P *< 0.05, Table 1). Four different tyrosine phosphorylated protein bands (approximately 18, 26, 34, and 36 kDa) increased after capacitation regardless of the litter size (*P *< 0.05, Fig. 1, Supplemental Materials, Fig. S1). However, there were no significant differences in tyrosine phosphorylation in high- and low-litter size spermatozoa before or after capacitation (Fig. 1).

### Proteomic analysis and identification of fertility-related proteins after capacitation

**Figure 2:**
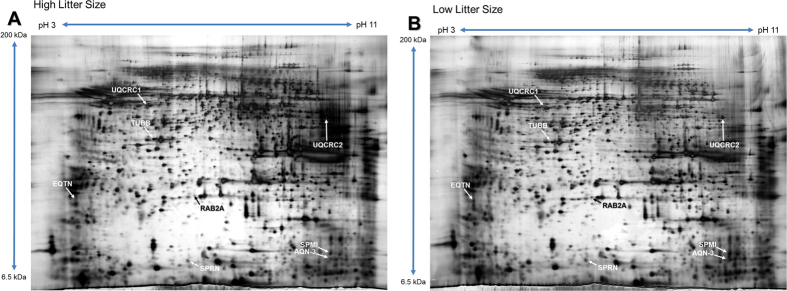
Separation of proteins with 2-DE. 2-DE gels were stained with silver nitrate and analyzed using PDQuest 8.0 software. (**A**) Protein spots from high-litter size spermatozoa after capacitation. (**B**) Protein spots from low-litter size spermatozoa after capacitation.

**Figure 3:**
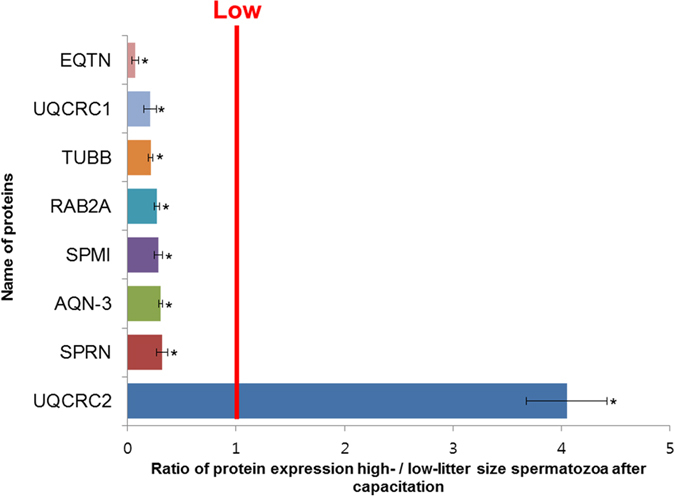
Comparison of proteins from high- and low-litter size spermatozoa after capacitation. Differentially expressed (>3-fold) proteins were identified by comparing high- and low-litter size spermatozoa (*P < 0.05). The line indicates equal expression in high- and low-litter size spermatozoa. The data represent the mean ± SEM, n = 3.

A total of 243 protein spots were detected, and 8 spots were significantly different in high- and low-litter size spermatozoa (>3-fold; *P *< 0.05; Fig. 2). Among them, 7 were stronger in the low-litter size spermatozoa, while 1 was stronger in the high-litter size spermatozoa (>3-fold; *P* < 0.05; Fig. 3). The differentially expressed proteins (>3-fold) were identified in an MS/MS ion search using MASCOT software. The 7 proteins abundantly expressed in the low-litter size spermatozoa were equatorin (EQTN), beta-tubulin (TUBB), UQCRC1, speriolin (SPRN), RAB2A, spermadhesin AQN-3 (AQN-3), and seminal plasma sperm motility inhibitor (SPMI). The protein abundantly expressed in the high-litter size spermatozoa was UQCRC2 (Table 2, Fig. 3).

### Protein confirmation by western blotting

**Figure 4:**
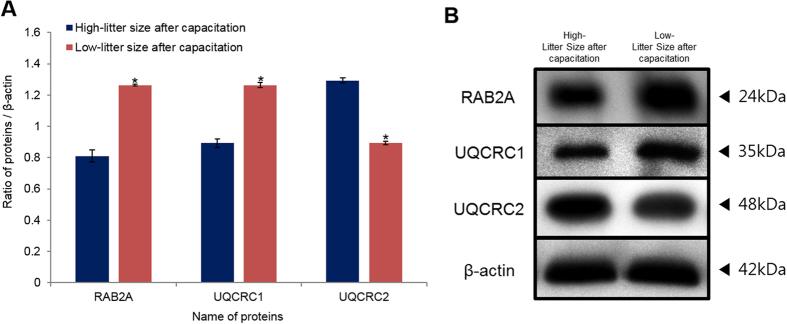
Expression of Ras-related protein Rab-2A (RAB2A), cytochrome b-c1 complex subunit 1 (UQCRC1), and cytochrome b-c1 complex subunit 2 (UQCRC2) in high- and low-litter size spermatozoa after capacitation. (**A**) Ratios of RAB2A, UQCRC1, and UQCRC2 (optical density [OD × mm]/β-actin [OD × mm]) in high- and low-litter size spermatozoa after capacitation (Navy bar: high-litter size after capacitation, Red bar: low-litter size after capacitation). Data represent the mean ± SEM, n = 3. Protein expression ratios denoted with an asterisk were significantly different (*P < 0.05). (**B**) RAB2A, UQCRC1, and UQCRC2 were probed with anti-RAB2A, anti-UQCRC1, and anti-UQCRC2 antibodies.

To validate the results, the differentially expressed proteins were examined with western blotting analysis using commercially available antibodies. RAB2A, UQCRC1, and UQCRC2 were detected at 24, 35, and 48 kDa, respectively. The proteins expression patterns were similar to those obtained with 2-DE. After capacitation, RAB2A and UQCRC1 were more abundant in low-litter size spermatozoa, and UQCRC2 was more abundant in high-litter size spermatozoa (*P *< 0.05, Fig. 4, Supplemental Materials, Fig. S2). In addition, RAB2A was abundantly expressed in low-litter size spermatozoa before capacitation (*P *< 0.05, Supplemental Materials, Fig. S3).

### Correlations between the expression of RAB2A, UQCRC1, and UQCRC2 and litter size

**Figure 5:**
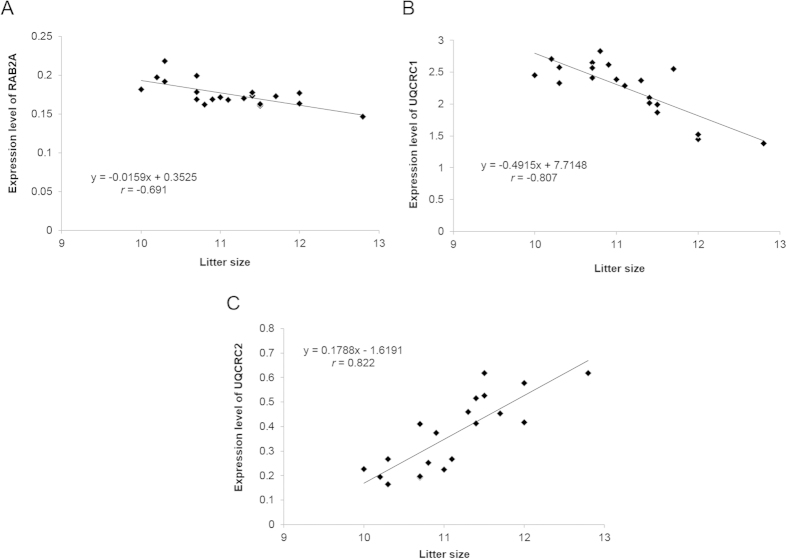
Relationship between litter size and proteins expression. (**A)** Significant correlations (*r* = −0.691, *P* < 0.01) were detected between litter size and RAB2A expression of 20 boars. (**B**) Significant correlations (*r* = −0.807, *P* < 0.01) were detected between litter size and RAB2A expression of 20 boars. (**C**) Significant correlations (*r* = 0.822, *P* < 0.01) were detected between litter size and RAB2A expression of 20 boars.

The expression of RAB2A, UQCRC1, and UQCRC2 in spermatozoa samples from 20 boars was measured with ELISA. To determine the correlation between the expression of RAB2A, UQCRC1, and UQCRC2 and litter size, Pearson correlation coefficients were calculated. RAB2A and UQCRC1 negatively correlated with litter size (*r* = −0.691 and −0.807, *P* < 0.01, Fig. 5). UQCRC2 positively correlated with litter size (*r* = 0.822, *P* < 0.01, Fig. 5).

### Quality assessment of parameters

**Figure 6:**
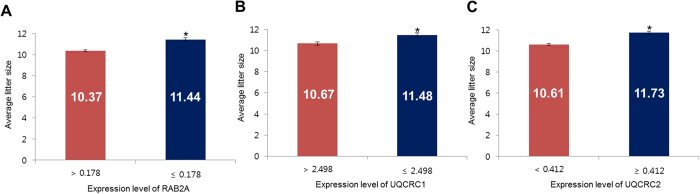
Average litter size according to the expression of ras-related protein Rab-2A (RAB2A), cytochrome b-c1 complex subunit 1 (UQCRC1), and cytochrome b-c1 complex subunit 2 (UQCRC2) in spermatozoa after capacitation. (**A**) Average litter size according to RAB2A expression. (**B**) Average litter size according to UQCRC1 expression. (**C**) Average litter size according to UQCRC2 expression.

To determine the cut-off value for litter size, a receiver operating curve (ROC) was used (Supplemental Materials, Fig. S4). According to the curve, the cut-off expression values for RAB2A (0.178), UQCRC1 (2.498), and UQCRC2 (0.412) corresponded to the maximal sensitivity and specificity. Therefore, these values were established as the lower limit (Table 3). For RAB2A expression, the sensitivity, specificity, negative predictive value, and positive predictive value were 100.00%, 66.67%, 100.00%, and 78.57%, respectively. The overall accuracy for the prediction of litters of 11 was 85.00% (Table 3, Supplemental Materials, Table S1). The average litter size of boars with RAB2A expression >0.178 was 10.37 piglets, whereas the average litter size of boars with RAB2A expression ≤0.178 was 11.44 piglets (*P* < 0.05, Fig. 6A). The sensitivity, specificity, negative predictive value, and positive predictive value of UQCRC1 were 90.90%, 88.89%, 88.89%, and 90.91%, respectively. The overall accuracy for the prediction of litters of ≥11 was 90.00% (Table 3, Supplemental Materials, Table S2). The average litter size of boars with UQCRC1 expression >2.498 was 10.67 piglets, whereas the average litter size of boars with UQCRC1 expression ≤2.498 was 11.48 piglets (*P* < 0.05, Fig. 6B). The sensitivity, specificity, negative predictive value, and positive predictive value of UQCRC2 were 81.80%, 100.00%, 81.82%, and 100.00%, respectively. The overall accuracy for the prediction of litters of ≥11 was 90.00% (Table 3, Supplemental Materials, Table S3). The average litter size of boars with UQCRC2 expression < 0.412 was 10.61 piglets, whereas the average litter size of boars with UQCRC2 expression ≥ 0.412 was 11.73 piglets (*P* < 0.05, Fig. 6C).

## Discussion

Proteomics research has provided a new way to study sperm-specific processes. Understanding these processes is important for identifying biomarkers of male fertility and for male contraceptive targeting. Several studies have reported the identification of fertility-related biomarkers in boar and bull spermatozoa^5^,^15^. However, new, more accurate techniques and biomarkers for the prognosis and diagnosis of male fertility are needed. Spermatozoa must undergo capacitation before fertilizing the oocyte *in vivo* and *in vitro*^12^,^13^,^14^,^16^,^17^,^18^. This fundamentally important event in fertilization is associated with changes that alter the protein content of the spermatozoa. Therefore, proteomic profiling of boar spermatozoa following capacitation might be helpful for improving the boar seed quality and attaining maximum reproductive competence.

In the present study, capacitation was induced in boar spermatozoa using mTCM 199 medium containing 10% fetal bovine serum and 10 μg/mL heparin. The method was also used by other researchers to induce capacitation in boar spermatozoa^9^,^16^,^19^,^20^. To validate the capacitation, we evaluated motion kinematics, morphological changes, and tyrosine phosphorylation in spermatozoa. Consistence with previous finding, our results demonstrated that incubation of boar spermatozoa with mTCM 199 significantly increased hyperactivation, curvilinear velocity, the mean amplitude of head lateral displacement, linearity, wobble, acrosome-reacted, and capacitated spermatozoa in both high- and low-litter size boar spermatozoa. Similar increased patterns of tyrosine phosphorylation also have been noticed in current study (Fig. 1). Interestingly, there were no significant differences of these parameters between high- and low-litter size spermatozoa. Therefore, the efficacy of the traditional methods to evaluate sperm quality is questionable.

To develop highly sensitive biomarkers for the evaluation of male fertility, comprehensive proteomic approaches were performed in high- and low-litter size spermatozoa after capacitation. Our result demonstrated that 8 proteins were differentially expressed between high- and low-litter size spermatozoa (>3-fold). UQCRC2 was highly expressed in high-litter size spermatozoa after capacitation, while EQTN, UQCRC1, TUBB, RAB2A, SPMI, AQN-3, and SPRN were highly expressed in low-litter size spermatozoa after capacitation. EQTN localizes to the equatorial segment in capacitated and acrosome-reacted spermatozoa. In addition, EQTN is exposed to the outside after the acrosome reaction; it then plays roles in sperm-oocyte interaction and fertilization^21^,^22^,^23^,^24^. Recent studies have demonstrated that EQTN is needed for the acrosome reaction but is not essential for acrosome formation^23^. An anti-EQTN monoclonal antibody inhibits mouse fertilization *in vivo*^25^. Thus, EQTN is a key protein in spermatozoa for the acrosome reaction, sperm-egg interaction, and fertilization. Tubulins are globular proteins that comprise a superfamily of five families (α-, β-, γ-, δ-, and ε-tubulin)^26^,^27^,^28^. α- and β-tubulin are major components of microtubules in spermatozoa^29^. A previous report postulated that TUBB plays a key role in sperm tail functions^30^. Audebert *et al.*^31^ demonstrated that inhibition of β-tubulin decreases the flagellar beat frequency by disrupting the tubulin-dynein head interaction. The finding indicates that β-tubulin is responsible for maintaining the hyperactivated motility in spermatozoa needed for capacitation. The RAB family has a critical role in regulating vesicular transport and membrane fusion. Earlier study suggested that RAB2A is located in the acrosome membrane in spermatozoa^32^,^33^. It was observed in head as well as tail of boar spermatozoa in present study (Supplemental Materials, Fig. S5). RAB2A modifies the structure of the acrosome to induce the acrosome reaction following capacitation^34^,^35^. SPMI, which inhibits sperm motility, is secreted exclusively from seminal vesicles and is rapidly degraded by prostatic proteases after ejaculation^36^,^37^,^38^. However, further research is needed to understand the effect of SPMI on spermatozoa. AQN-3, a ZP-binding protein, has been identified in low-molecular mass in boar spermatozoa^39^. Previous studies have reported that high amounts of this protein coat the sperm surface after ejaculation; the proteins are released after capacitation^40^. These studied suggest that AQN-3 plays a key role in capacitation and gamete recognition^40^. SPRN, a spermatogenic cell-specific centrosomal protein, localizes to the flagellum of condensing spermatids^41^. Studies have demonstrated that abnormal sperm centrioles result in defects in sperm motility in infertile males^42^,^43^,^44^,^45^. Therefore, abnormalities in SPRN cause infertility by inducing abnormalities in the sperm centrosome^46^.

Interestingly, although sperm parameters (motility, motion kinematics, capacitation status, and tyrosine phosphorylation levels) were not significantly different between high- and low-litter size spermatozoa, EQTN, TUBB, RAB2A, SPMI, AQN-3, and SPRN exhibited distinct differences in expression according to litter size. However, there are no studies about this issue following capacitation. Therefore, our results might provide insight into sperm function and the mechanism of fertilization. These proteins might also be useful biomarkers for the diagnosis and prognosis of male fertility.

UQCRC1 and UQCRC2, subunits of the respiratory chain protein ubiquinol cytochrome c reductase, are associated with oxidative stress and the generation of reactive oxygen species (ROS) in mitochondria^47^,^48^,^49^. Both proteins were located in the head and tail in boar spermatozoa (Supplemental Materials, Fig. S5). A recent study reported that ubiquinol cytochrome c reductase regulates tyrosine phosphorylation during capacitation and the acrosome reaction in boar spermatozoa^50^. Aguilera-Aguirre *et al.*^48^ have shown that UQCRC2 deficiency induces ROS production. Other studies have reported that UQCRC2 expression correlates with male fertility^5^,^51^. In the present study, the patterns of UQCRC1 and UQCRC2 expression in high- and low-litter size spermatozoa after capacitation were evaluated using a proteomics approach. Of note, a recent study reported that UQCRC2 is highly expressed in bull spermatozoa with low fertility^5^. However, the study focused on freeze-thawed semen without capacitation^5^. Interestingly, the difference of expression levels of both proteins was non-significant in high- and low-litter size boar spermatozoa before capacitation (Supplemental Materials, Fig. S3). Therefore, additional studies of UQCRC1 and UQCRC2 in spermatozoa following capacitation are needed to understand the mechanism by which UQCRC1 and UQCRC2 affect male fertility.

RAB2A and UQCRC1 negatively correlated with litter size (*P* < 0.01), while UQCRC2 positively correlated with litter size (*P* < 0.01). The overall accuracy of the assay in predicting litter size using the expression level of RAB2A, UQCRC1, and UQCRC2 was 85.00%, 90.00%, and 90.00%, respectively (Table 3). The protein expression levels, using cut-off values for litters of 11, were able to differentially predict litter sizes that differed by 1 pup in AI field trials (Fig. 6). The results suggest that differentially expressed proteins can be used to predict litter size with high accuracy in the swine industry. Several studies have reported that fertility-related biomarkers identified with proteomics can be used for the diagnosis and prognosis of male fertility in economic animals. Park *et al.*^5^ identified a set of fertility-related proteins in bull spermatozoa and validated the protein markers with western blotting. They found that enolase 1, voltage dependent anion channel 2, and UQCRC2 correlated with individual fertility. However, the accuracy of these markers was not described. More recently, Kwon *et al.*^15^ identified several fertility-related biomarkers in boar spermatozoa and evaluated the accuracy of these biomarkers in field trials. They reported that RAB2A, triosephosphate isomerase (TPI), NADH dehydrogenase [ubiquinone] iron-sulfur protein 2 (NDUFS2), calmodulin (CALM), and mitochondrial malate dehydrogenase 2 (MDH2) correlated with litter size. The average overall accuracy was 73% (RAB2A = 70%, TPI = 60%, NDUFS2 = 70%, CALM = 85%, MDH2 = 80%). However, the average overall accuracy of fertility-related biomarkers identified in the current study was more than 88%.

The expression of RAB2A in high- and low-litter size spermatozoa after capacitation was similar with previously reported study^15^. Interestingly, the overall accuracy of RAB2A expression in predicting litter size was 15% higher than previous study^15^. Moreover, in field trials, RAB2A predicted an average litter size that was increased by 1 pup, compared with 0.8 pups in the study by Kwon *et al.*^15^. Therefore, we anticipate that the fertility-related biomarkers identified in the present study will be sensitive biomarkers for the prognosis and diagnosis of male fertility.

Taken together, our results suggest that protein biomarkers offer a new, more accurate tool for predicting superior sires in the economic animal industry. However, ELISA method to monitor expression levels of target proteins could be less reliable since the analysis was performed on 20 boars spermatozoa at one time point. Therefore, further studies are required to validate whether expression level of target protein is stable in time to better elucidate the possible clinical applications of the discovered protein marker.

## Materials and Methods

Landrace semen samples and each male pig’s fertility field data (total pups/total breedings) were supplied by Sunjin Co. (Danyang, Korea). To eliminate fertility variation following parity, parity 1 fertility data was excluded and only parity 2–8 fertility data was included^15^. Nine high-litter size (12.8 ± 0.09) semen samples and 9 low-litter size (10.19 ± 0.19) semen samples were collected. High- and low-litter size semen samples were randomly divided into 3 groups each for experimental replication (n = 3, Supplemental Materials, Fig. S6). To rule out individual variation, each group’s semen samples were mixed. Pooled samples were processed at 500 × *g* for 20 min with a discontinuous (70% [v/v] and 35% [v/v]) Percoll gradient (Sigma, St. Louis, MO, USA) to remove seminal plasma and dead spermatozoa^20^. For the ELISA, samples were collected from randomly selected 20 boars (known fertility field data). Then the samples were also washed in same manner. To induce capacitation, samples were incubated with modified tissue culture medium (mTCM) 199 (containing 10% fetal bovine serum [v/v], 0.91 mM sodium pyruvate, 3.05 mM d-glucose, 2.92 mM calcium lactate, 2.2 g/L sodium bicarbonate, and 10 μg/mL heparin) (Sigma) for 30 min at 37 °C under an atmosphere of 5% CO_2_ in air^9^,^16^,^19^,^20^. All procedures were performed according to guidelines for the ethical treatment of animals and were approved by the Institutional Animal Care and Use Committee of Chung-Ang University.

### Computer-assisted sperm analysis

To analyze the motility and motion kinematics of before-capacitation samples, the samples were pre-incubated with mTCM 199 (without 10% fetal bovine serum [v/v] and 10 μg/mL heparin) for 10 min at 37 °C under an atmosphere of 5% CO_2_ in air. The after-capacitation samples were analyzed following a 30-min incubation in the capacitation medium described earlier. A CASA system (SAIS-PLUS v.10.1; Medical Supply, Seoul, Korea) was used to analyze sperm motility (%) and motion kinematics. Briefly, 10 μL of sample was placed in a Makler chamber (Makler, Haifa, Israel). The filled chamber was placed on a stage preheated to 37 °C. Using a 10× objective in phase contrast mode, the image was relayed, digitized, and analyzed using the SAIS-PLUS software. The movement of at least 250 sperm cells was recorded for each sample from more than five randomly selected fields per replicate.

### H33258/CTC assessment of capacitation status

The capacitation status was determined using the dual staining method described by Kwon *et al.*^12^,^13^,^52^. Briefly, 135 μL of treated spermatozoa was added to 15 μL of H33258 solution (10 μg H33258/mL Dulbecco’s PBS [DPBS]) and incubated for 2 min at room temperature (RT). Excess dye was removed by layering the mixture over 250 μL of 2% (w/v) polyvinylpyrrolidone in DPBS. After centrifugation at 100 × *g* for 2.5 min, the supernatant was discarded, and the pellet was resuspended in 100 μL of DPBS. Thereafter, 100 μL of a freshly prepared CTC solution (750 mM CTC in 5 μL buffer: 20 mM Tris, 130 mM NaCl, and 5 mM cysteine, pH 7.4) was added. Samples were viewed with a Microphot-FXA microscope (Nikon) under epifluorescence illumination using ultraviolet BP 340–380/LP 425 and BP 450–490/LP 515 excitation/emission filters for H33258 and CTC, respectively. The spermatozoa were classified as live non-capacitated (F, bright green fluorescence distributed uniformly over the entire sperm head, with or without a stronger fluorescent line at the equatorial segment), live capacitated (B, green fluorescence over the acrosomal region and a dark post-acrosomal region), or live acrosome-reacted (AR, sperm showing a mottled green fluorescence over the head, green fluorescence only in the post-acrosomal region, or no fluorescence over the head)^12^,^13^. Two slides, with at least 400 spermatozoa per slide, were evaluated per sample.

### Two-dimensional gel electrophoresis and gel image analysis

Proteins were extracted and analyzed using 2-DE and ESI-MS/MS. For protein extraction, spermatozoa (50 × 10^6^) were incubated in rehydration buffer containing 7 M urea (Sigma), 2 M thiourea (Sigma), 4% (w/v) 3-[(3-cholamidopropyl)dimethylammonio]-1-propanesulfonate (USB), 0.05% (v/v) Triton X-100 (Sigma), 1% (w/v) octyl β-d-glucopyranoside, 24 μM PMSF (Sigma), 1% (w/v) DTT (Sigma), 0.5% (v/v) IPG buffer, and 0.002% (w/v) bromophenol blue at 4°C for 1 h. Thereafter, 250 μg of solubilized protein from the sperm cells in 450 μL of rehydration buffer was placed in a rehydration tray with 24-cm-long NL Immobiline DryStrips (pH 3–11; Amersham, Piscataway, NJ, USA) for 12 h at 4°C. First dimension electrophoresis was performed using an IPGphor isoelectric focusing apparatus (Amersham). The strips were then focused at 100 V for 1 h, 200 V for 1 h, 500 V for 1 h, 1000 V for 1 h, 5000 V for 1.5 h, 8000 V for 1.5 h, and 8000–90,000 V for 1 h. After isoelectric focusing, the strips were equilibrated with equilibration buffer A containing 6 M urea, 75 mM Tris-HCl (pH 8.8), 30% (v/v) glycerol, 2% (w/v) SDS, 0.002% (w/v) bromophenol blue, and 2% (w/v) DTT for 15 min at RT. The strips were equilibrated a second time with equilibration buffer B (equilibration buffer A with 2.5% (w/v) iodoacetamide (Sigma) but without DTT) for 15 min at RT. Second-dimension electrophoresis was carried out with 12.5% (w/v) SDS-PAGE gels with the strips at 100 V for 1 h and 500 V until the bromophenol blue front began to migrate off the lower end of the gel. The gels were silver-stained for image analysis according to the manufacturer’s instructions (Amersham) and scanned using a high-resolution GS-800 calibrated scanner (Bio-Rad, Hercules, CA, USA). The detected spots were matched, and high- and low-litter size spermatozoa gels were compared using PDQuest 8.0 software (Bio-Rad). The low-litter size spermatozoa gel was used as the control. Finally, the density of the spots was calculated and normalized as the ratio of other gel/low-litter size spermatozoa gel.

### Protein identification

Proteins identification was performed as described previously^15^,^16^. Excised gel spots were destained in 100 μL of destaining solution (30 mM potassium ferricyanide, 100 mM sodium thiosulfate) with shaking for 5 min. The solution was removed, and the gel spots were incubated with 200 mM ammonium bicarbonate for 20 min. The gel pieces were dehydrated with 100 μL of acetonitrile and dried in a vacuum centrifuge. The dried gel pieces were rehydrated with 20 μL of 50 mM ammonium bicarbonate containing 0.2 μg modified trypsin (Promega) for 45 min on ice. The solution was removed, and 30 μL of 50 mM ammonium bicarbonate was added. The peptide solution was desalted using a C18 nano column (homemade). A column consisting of 100–300 nL of POROS reversed phase R2 material (20–30 μm bead size; PerSeptive Biosystems) was packed in a constricted GELoader tip (Eppendorf). Thirty microliters of the peptide mixture from the digestion supernatant was diluted in 30 μL of 5% formic acid, loaded onto the column, and washed with 30 μL of 5% formic acid. For MS/MS analyses, the peptides were eluted with 1.5 μL of 50% methanol/49% H_2_O/1% formic acid directly into a pre-coated borosilicate nano-electrospray needle (EconoTip; New Objective).

MS/MS of the peptides generated by in-gel digestion was performed using a nano-ESI on a Q-TOF2 mass spectrometer (AB Sciex Instruments). A potential of 1 kV was applied to the pre-coated borosilicate nano-electrospray needles in the ion source, combined with a nitrogen back-pressure of 0–5 psi to produce a stable flow rate (10–30 nL/min). The cone voltage was 40 V. The collision gas was argon at a pressure of 6^–7^ × 10^–5^ mbar, and the collision energy was 25–40 V. Product ions were analyzed using an orthogonal TOF analyzer that was fitted with a reflector, a micro-channel plate detector, and a time-to-digital converter. “MS/MS ion search” was assigned as the ion search option in Mascot 2.4 software and a Swiss-Prot FASTA. The peptide fragment files were searched based on the database using the Mascot search DB [NCBInr (May 2014)] and Homology search DB [UniprotKB/TrEMBL, UniprotKB/Swissprot(Oct 2014)] using the MASCOT search engine (Matrix Science). The mass tolerance was set at ±1 and ±0.6 Da for the peptides and fragments, respectively. High-scoring peptides corresponded to peptides that were above the default significance threshold in MASCOT (*P* < 0.05, peptide score > 35).

### Western blotting

To evaluate the capacitation status, tyrosine phosphorylated proteins were detected with an anti-phosphotyrosine antibody. To confirm the 2-DE results, western blotting bands, visualized using antibodies against RAB2A, UQCRC1 and UQCRC2, were quantified in three different pooled spermatozoa of high- and low-litter size spermatozoa sample groups (randomly divided 3 groups) before and after capacitation. Western blotting was performed as described previously^15^,^16^ with modifications. The samples were washed twice with DPBS and centrifuged at 10,000 × *g* for 5 min. The pellets were then resuspended and incubated with sample buffer containing 5% 2-mercaptoethanol for 10 min at RT. After incubation, the insoluble fractions were separated by centrifugation at 10,000 × *g* for 10 min. The samples were subjected to SDS-PAGE using a 12% mini-gel system (Amersham), and the separated proteins were transferred to PVDF membranes (Amersham). The membranes were blocked with 3% blocking agent (Amersham) for 1 h at RT. Tyrosine phoaphorylated proteins, RAB2A, UQCRC1, and UQCRC2 in high- and low-litter size spermatozoa after capacitation were immunodetected using the anti-phosphotyrosine and anti-UQCRC2 mouse polyclonal antibodies and anti-RAB2A and anti-UQCRC1 rabbit polyclonal antibodies (Amersham) diluted with blocking solution (1 μg/mL), After 2 h at RT, the membranes were washed and incubated for 1 h at RT with horse radish peroxidase (HRP)-conjugated goat anti-mouse IgG and anti-rabbit IgG (Abcam) diluted with 3% blocking agent (1:5000). The membranes were washed 3 times with DPBS containing 0.1% Tween-20. The proteins on the membranes were detected using ECL reagents. Proteins on the membranes were stripped with membrane stripping solution (2% SDS, 100 mM mercaptoethanol, and 62 mM Tris-HCl) after detection. β-actin was then detected by incubating the membranes for 2 h at RT with an HRP-conjugated monoclonal anti-β-actin mouse antibody (Abcam) diluted with blocking solution (1:10,000). β-actin was detected using ECL reagents. All of the bands were scanned with a GS-800 calibrated imaging densitometer (Bio-Rad) and analyzed with Quantity One software (Bio-Rad). The signal intensity ratios of the tyrosine phoaphorylated proteins, RAB2A, UQCRC1, and UQCRC2 bands were calculated relative to the intensity of the β-actin band.

### Enzyme-linked immunosorbent assay (ELISA)

To determine the correlation between RAB2A, UQCRC1, and UQCRC2 and litter size in boar, ELISA was performed using spermatozoa from 20 boars. For protein extraction, spermatozoa samples (50 × 10^6^/mL) were incubated in rehydration buffer containing 7 M urea (Sigma), 2 M thiourea (Sigma), 4% (w/v) 3-[(3-cholamidopropyl) dimethylammonio]-1-propanesulfonate (USB), 0.05% (v/v) Triton X-100 (Sigma), 1% (w/v) octyl β-d-glucopyranoside, 24 μM PMSF (Sigma), 1% (w/v) DTT (Sigma), and 0.002% (w/v) bromophenol blue at 4 °C for 1 h. Purified protein (50 μg/well) was coated onto 96-well Immuno Plates (Thermo Scientific, Waltham, MA, USA) and incubated overnight at 4 °C. The plates were blocked with blocking solution (5% [w/v] BSA in DPBS containing 0.5% Tween-20 for 90 min at 37 °C. The plates were then incubated for 90 min at 37 °C with anti-RAB2A and anti-UQCRC1 rabbit polyclonal antibodies and anti-UQCRC2 mouse polyclonal antibody (Amersham) diluted with blocking solution (1:5000). The plates were incubated for 90 min at 37 °C with HRP-conjugated anti-mouse and anti-rabbit IgG (Abcam) diluted with blocking solution (1:5000). To activate peroxidase, the plates were incubated with TMB solution (Sigma) for 15 min at RT, and the reaction was stopped with 1 N sulfuric acid. The signal was measured at 450 nm using a microplate reader (Gemini EM; Molecular Devices Corporation).

### Quality assessment of parameters

Four key parameters were used in the screening tests: sensitivity, specificity, positive predictive value, and negative predictive value^9^,^15^,^19^. Sensitivity was determined as the percentage of boars whose litter size was correctly identified by the test. Specificity was determined as the percentage of boars that tested truly negative. The positive predictive value was determined as the percentage of boars that tested positive and actually had a litter size ≥11 or <11. The negative predictive value was determined as the percentage of boars that tested negative and actually had a litter size of ≥11 or <11.

### Statistical analysis

The data were analyzed in SPSS (v.18.0; Chicago, IL, USA). Pearson correlation coefficients were calculated to determine the association between the expression of RAB2A, UQCRC1, and UQCRC2 and litter size. ROCs were used to assess the ability of the individual analyzed parameters to predict a litter size of ≥11 or <11 (based on average litter size). The cut-off value, calculated from the ROCs, was determined in relation to the point that maximized specificity and sensitivity^9^,^15^,^19^. Student’s two-tailed *t*-test was used to compare protein expression and litter size predicted by the ROCs. *P* < 0.05 was considered significantly different. All data were expressed as the mean ± SEM.

## Additional Information

**How to cite this article**: Kwon, W.-S. *et al.* Increased male fertility using fertility-related biomarkers. *Sci. Rep.* **5**, 15654; doi: 10.1038/srep15654 (2015).

## Supplementary Material

Supplementary Information

## Figures and Tables

**Table 1:** Sperm motility, motion kinematics, and capacitation status in high- and low-litter size Landrace spermatozoa following capacitation.

Sperm motility,motion kinematics,and capacitation status	High-litter sizespermatozoa beforecapacitation	High-litter sizespermatozoa aftercapacitation	Low-litter sizespermatozoa beforecapacitation	Low-litter sizespermatozoa aftercapacitation
MOT (%)	86.31 ± 0.37	86.95 ± 0.58	85.42 ± 0.88	85.20 ± 1.60
HYP (%)	5.35 ± 2.15	15.94 ± 0.76*	7.31 ± 1.02	13.54 ± 0.62*
VCL (μm/s)	122.89 ± 5.84	153.37 ± 3.85*	130.33 ± 1.55	147.95 ± 0.73*
VSL (μm/s)	73.57 ± 0.68	75.50 ± 6.74	67.81 ± 2.64	74.77 ± 6.32
VAP (μm/s)	77.70 ± 1.15	86.77 ± 3.98	75.61 ± 2.35	86.20 ± 3.65
ALH (μm)	5.71 ± 0.23	6.98 ± 0.23*	5.95 ± 0.09	6.81 ± 0.12*
LIN (%)	59.52 ± 2.85	46.73 ± 1.74*	53.66 ± 0.82	47.50 ± 1.74*
WOB (%)	63.40 ± 2.08	56.52 ± 1.04*	58.65 ± 0.62	54.24 ± 1.24*
AR (%)	3.16 ± 0.42	11.52 ± 1.90*	1.53 ± 0.02	10.95 ± 2.19*
F (%)	87.04 ± 0.62	46.78 ± 2.71*	89.60 ± 3.41	49.22 ± 4.31*
B (%)	9.80 ± 0.20	41.70 ± 1.22*	8.87 ± 3.39	39.84 ± 3.05*

Sperm motility, motion kinematics, and capacitation status are presented as the mean ± SEM, n = 3. *P < 0.05. MOT = motility (%); VCL = curvilinear velocity (μm/s); VSL = straight-line velocity (μm/s); VAP = average path velocity (μm/s); ALH = mean amplitude of head lateral displacement (μm); LIN (%) = linearity; WOB (%) = wobble; AR = live acrosome-reacted pattern (%); F = live non-capacitated pattern (%); B = live capacitated pattern (%).

**Table 2:** Differentially expressed (>3-fold) proteins identified with ESI-MS/MS.

**GI no.**	**Symbol**	**Protein description**	**Peptide sequence**	Mascotscore*
gi|31124567	EQTN	Equatorin	R.ATTDLNFSLR.N	60
gi|135490	TUBB	Beta-tubulin	R.IMNTFSVVPSPK.V	55
gi|33528584	UQCRC1	Cytochrome b-c1 complex subunit 1	R.RIPLAEWESR.I R.ADLTEYVSQHYK.A R.NALVSHLDGTTPVCEDIGR.SK.HFSSLSGTYVEDAVPAFTPCR.F K.AVELLADIVQNCSLEDSQIEK.EK.AVELLADIVQNCSLEDSQIEKER.D	207
gi|545819718	SPRN	Speriolin	R.ILSGIFPER.V R.LYGFTVSNIPEK.I R.ERPELAASEGGSYTVDFLQR.V	106
gi|31125379	RAB2A	Ras-related protein Rab-2A	K.LQIWDTAGQESFR.S	55
gi|114083	AQN-3	Spermadhesin AQN-3	F.VYQSSHNVATVK.Y	68
gi|12585344	SPMI	Seminal plasma sperm motility inhibitor	F.VYQSSSNVATVK.Y	68
gi|335284501	UQCRC2	Cytochrome b-c1 complex subunit 2	R.LPNGLVIASLENYAPASR.I K.ALASAAPAGVPLQPQDLEFTR.L K.AVAFQNPQAQVLENLHAAAYR.N	69

^*^The MASCOT score is −10 log (*P*), where *P* is the probability that the observed match is a random event. Individual scores of >50 indicate identity or extensive homology (*P* < 0.05).

**Table 3:** Correlation between the expression of Ras-related protein Rab-2A (RAB2A), cytochrome b-c1 complex subunit 1 (UQCRC1), and cytochrome b-c1 complex subunit 2 (UQCRC2) and litter size.

	Sensitivity(%)	Specificity(%)	Negativepredictive value(%)	Positivepredictive value(%)	Overallaccuracy(%)
RAB2A	100.00	66.67	100.00	78.57	85.00
UQCRC1	90.91	88.89	88.89	90.91	90.00
UQCRC2	81.82	100.00	81.82	100.00	90.00
