# Supplementary material for: Increased male fertility using fertility-related biomarkers
Source: Sci Rep. 2015 Oct 22;5:15654. doi: 10.1038/srep15654 (PMC4614854; doi:10.1038/srep15654)

**Supplemental Materials**

**Kwon *et al*.**

**Increased male fertility using fertility-related biomarkers**

Woo-Sung Kwon, Md Saidur Rahman, Do-Yeal Ryu, Yoo-Jin Park, and Myung-Geol Pang^*^

Department of Animal Science & Technology, Chung-Ang University, Anseong, Gyeonggi-Do 456-756, Korea

^*^Correspondence *E-mail*: [mgpang@cau.ac.kr](mailto:mgpang@cau.ac.kr); Tel: +82.31.670.4841; Fax: +82.31.675.9001

**Table S1. Correlation between expression level of RAB2A and litter size.**

|  | Litter size ≥ 11 | Litter size < 11 |
| --- | --- | --- |
| Expression level of RAB2A ≤ 0.178 (n = 14) | 11^A^ | 3^B^ |
| Expression level of RAB2A > 0.178 (n = 6) | 0^C^ | 6^D^ |
|  |  | |
| Sensitivity | 100.00 | |
| Specificity | 66.67 | |
| Negative predictive value | 100.00 | |
| Positive predictive value | 78.57 | |
| Overall accuracy | 85 | |

Sensitivity = [A/(A + C)] × 100; Specificity = [D/(B + D)] × 100; Positive predictive value = [A/(A + B)] × 100, negative predictive value = [C/(C + D)] × 100, and overall accuracy = [(A + D)/(A + B + C + D)] × 100.

**Table S2. Correlation between expression level of UQCRC1 and litter size.**

|  | Litter size ≥ 11 | Litter size < 11 |
| --- | --- | --- |
| Expression level of UQCRC1 ≤ 2.498 (n = 11) | 10^A^ | 1^B^ |
| Expression level of UQCRC1 > 2.498 (n = 9) | 1^C^ | 8^D^ |
|  |  | |
| Sensitivity | 90.91 | |
| Specificity | 88.89 | |
| Negative predictive value | 88.89 | |
| Positive predictive value | 90.91 | |
| Overall accuracy | 90.00 | |

Sensitivity = [A/(A + C)] × 100; Specificity = [D/(B + D)] × 100; Positive predictive value = [A/(A + B)] × 100, negative predictive value = [C/(C + D)] × 100, and overall accuracy = [(A + D)/(A + B + C + D)] × 100.

**Table S3. Correlation between expression level of UQCRC2 and litter size.**

|  | Litter size ≥ 11 | Litter size < 11 |
| --- | --- | --- |
| Expression level of UQCRC2 ≥ 0.412 (n = 9) | 9^A^ | 0^B^ |
| Expression level of UQCRC2 < 0.412 (n = 11) | 2^C^ | 9^D^ |
|  |  | |
| Sensitivity | 81.82 | |
| Specificity | 100.00 | |
| Negative predictive value | 81.82 | |
| Positive predictive value | 100.00 | |
| Overall accuracy | 90.00 | |

Sensitivity = [A/(A + C)] × 100; Specificity = [D/(B + D)] × 100; Positive predictive value = [A/(A + B)] × 100, negative predictive value = [C/(C + D)] × 100, and overall accuracy = [(A + D)/(A + B + C + D)] × 100.

**Fig. S1 The blot images for Fig. 1 (all three replicates).** The western blot analysis was conducted under same experimental condition as indicated materials and methods.

**
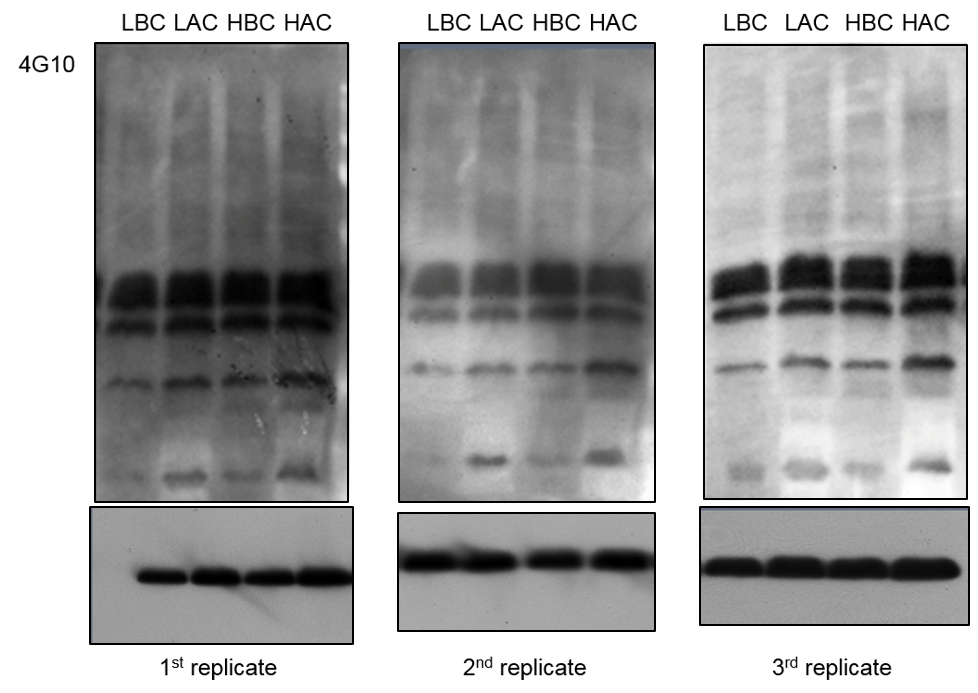
**

**Fig. S2 The uncropped blot images for Fig. 4.** The western blot analysis was conducted under same experimental condition as indicated materials and methods.

**
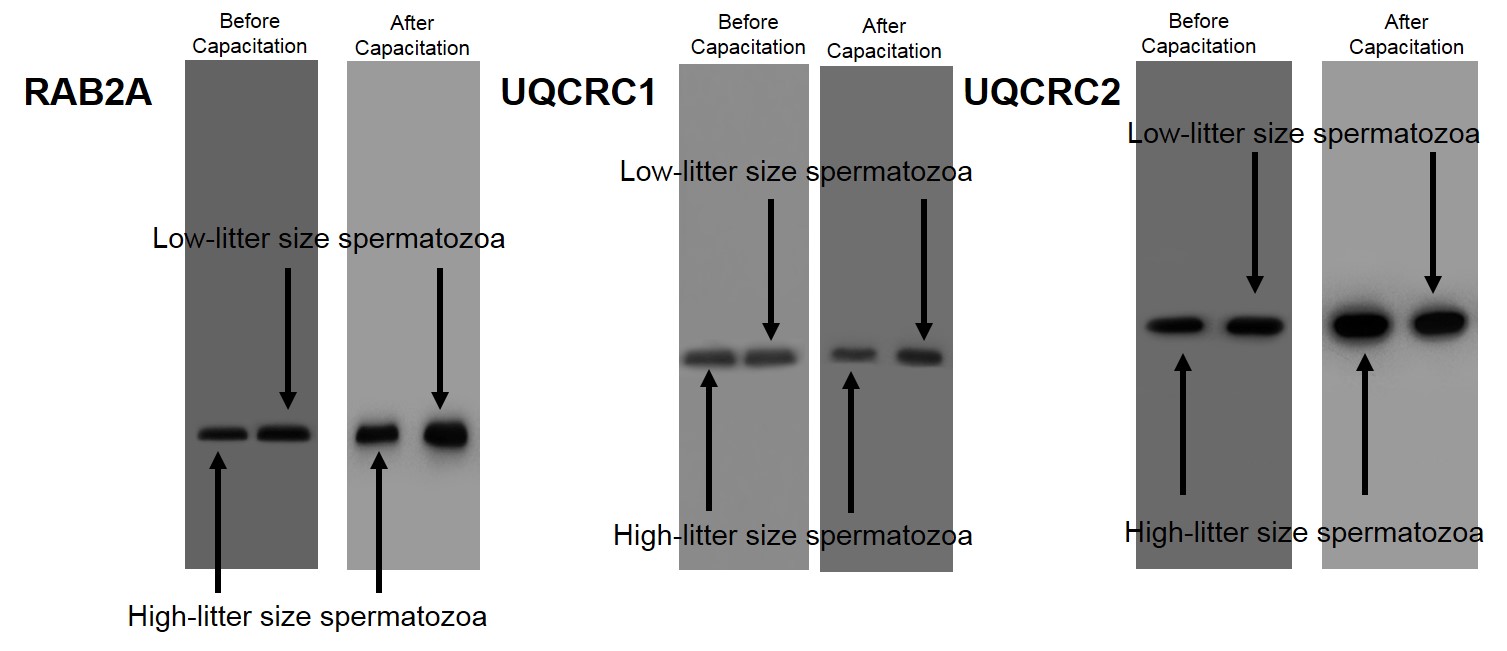
**

**Fig. S3 Expression of Ras-related protein Rab-2A (RAB2A), cytochrome b-c1 complex subunit 1 (UQCRC1), and cytochrome b-c1 complex subunit 2 (UQCRC2) in high- and low-litter size spermatozoa before capacitation.** (A) Ratios of RAB2A, UQCRC1, and UQCRC2 (optical density [OD × mm]/β-actin [OD × mm]) in high- and low-litter size spermatozoa before capacitation (Navy bar: high-litter size before capacitation, Red bar: low-litter size before capacitation). Data represent the mean ± SEM, n = 3. Protein expression ratios denoted with an asterisk were significantly different (**P* < 0.05). (B) RAB2A, UQCRC1, and UQCRC2 were probed with anti-RAB2A, anti-UQCRC1, and anti-UQCRC2 antibodies.


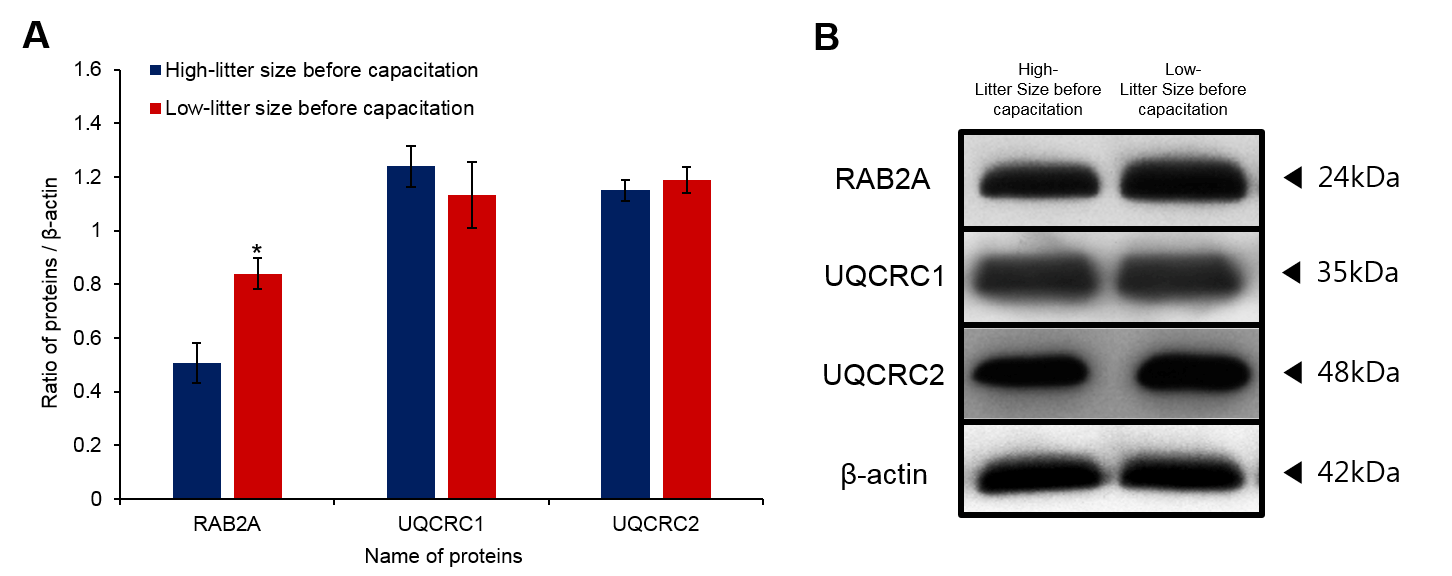


**Fig. S4 Receiver operating characteristic (ROC) curves for various expression levels of proteins.** (A) ROC curve for various expression levels of RAB2A. (B) ROC curve for various expression levels of UQCRC1. (C) ROC curve for various expression levels of UQCRC2.

**
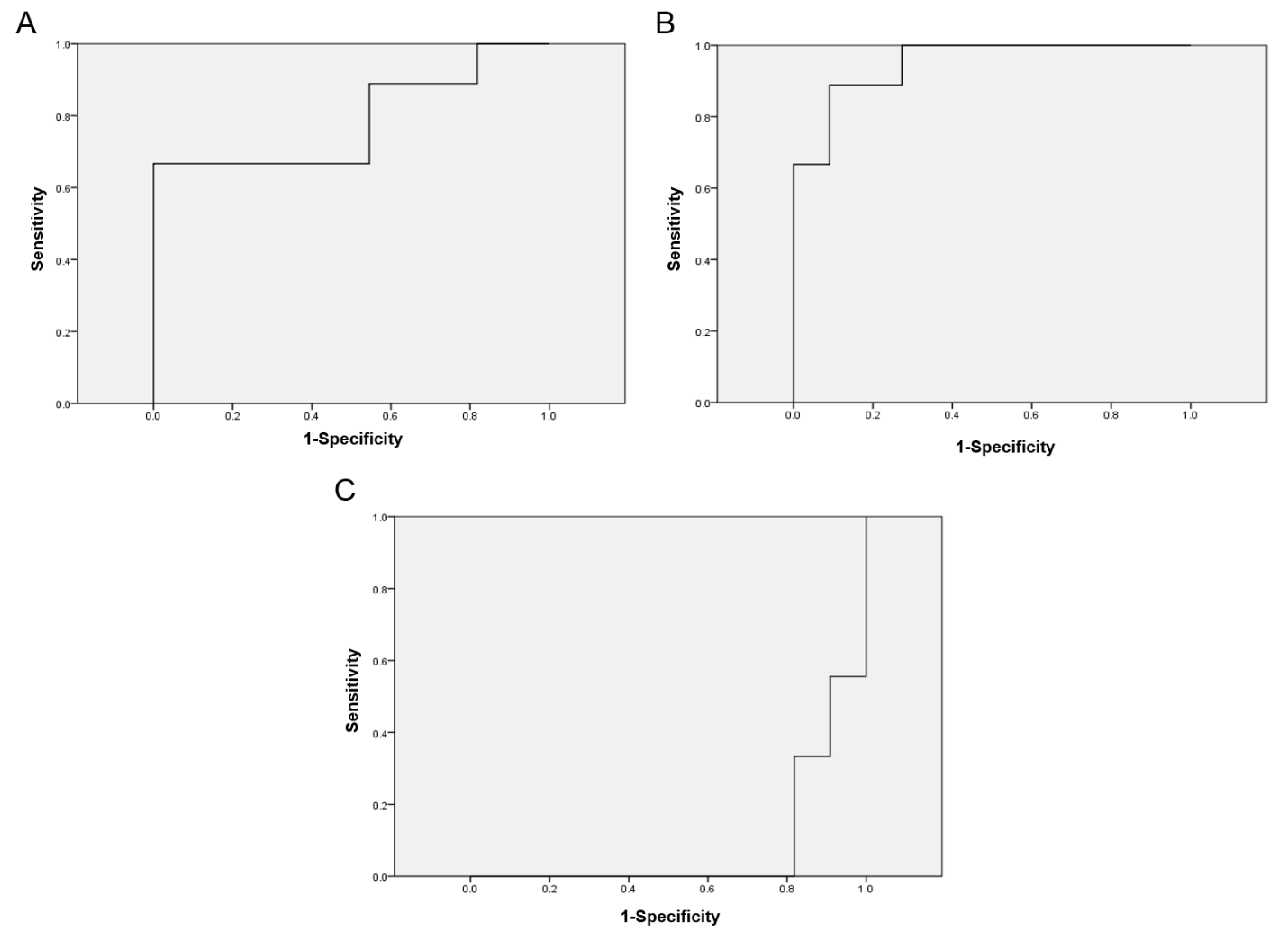
**

**Fig. S5 Localization of RAB2A, UQCRC1 and UQCRC2 in boar spermatozoa before- and after-capacitation.** (A, C, and E) Images of RAB2A, UQCRC1 and UQCRC2 before-capacitation (green). (B, D and F) Images of RAB2A, UQCRC1 and UQCRC2 after-capacitation (green). (G-L) Merged images of nucleus (DAPI, blue) and acrosome (lectin PNA, red). (M, O and Q) Merged images of nucleus (DAPI, blue), acrosome (lectin PNA, red) and reactivity profile with antibodies (green) against RAB2A, UQCRC1 and UQCRC2 before capacitation, respectively. (N, P and R) Merged images of nucleus (DAPI, blue), acrosome (lectin PNA, red) and reactivity profile with antibodies (green) against RAB2A, UQCRC1 and UQCRC2 after capacitation, respectively. Images were obtained using a Nikon TS-1000 microscope and NIS Elements image software (Nikon, Japan). Bar = 20μm.


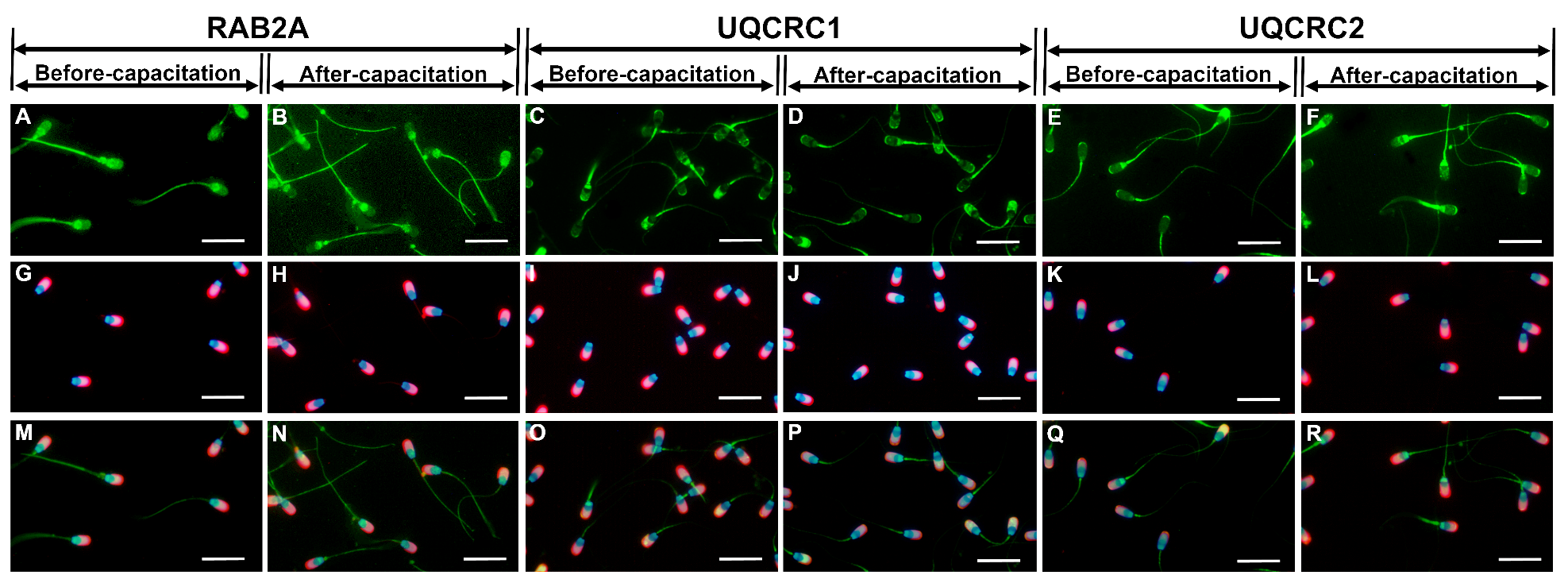


**Fig. S6** **The average litter size of high- and low-litter size spermatozoa.** The data represent the mean ± S.E., *n* = 3. * was significantly different between high- and low-litter size spermatozoa by Student’s two-tailed t-test (*P* < 0.01).


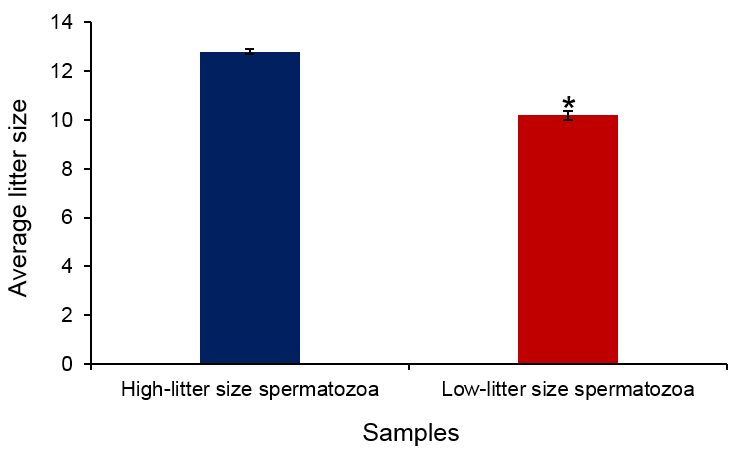

Supplement: Supplementary Information [file srep15654-s1.doc]
